# Supramolecular interaction enabled preparation of high-strength water-based adhesives from polymethylmethacrylate wastes

**DOI:** 10.1016/j.isci.2023.106022

**Published:** 2023-01-23

**Authors:** Jing Kang, Xiang Li, Yunlu Zhou, Ling Zhang

**Affiliations:** 1 State Key Laboratory of Supramolecular Structure and Materials, College of Chemistry, Jilin University, Changchun 130012, P. R. China

**Keywords:** Chemistry, Chemical engineering, Supramolecular chemistry

## Abstract

The preparation of water-based adhesives with high bonding strength for various substrates is challenging. Moreover, to construct a sustainable society, it is highly desirable to develop a cost-effective way to achieve the reuse of plastic wastes. Herein, using polymethylmethacrylate (PMMA) chemicals or wastes as raw materials, water-based adhesives with high bonding strength for various substrates are prepared through a simple one-step hydrolysis strategy. The adhesives possess the maximum bonding strength of 7.1 MPa to iron, 4.2 MPa to wood, and ∼1.5 MPa to plastics. The adhesives have a world-record bonding strength to metal when compared with that of current reported water-based adhesives. Our method is low cost, simple, environmentally friendly, and suitable for large-scale industrial production. More importantly, using plastic wastes as raw materials opens up a new and low-cost way to turn wastes into valuables, which will greatly contribute to construct a sustainable society.

## Highlights

• High-strength, water-based adhesives suitable for various substrates were developed
• Polymethylmethacrylate wastes can be used as raw materials for adhesive preparation
• The adhesives have a world-record bonding strength to metal substrates
• Preparation method is low cost, and suitable for large-scale industrial production

## Graphical abstract

**Figure:**
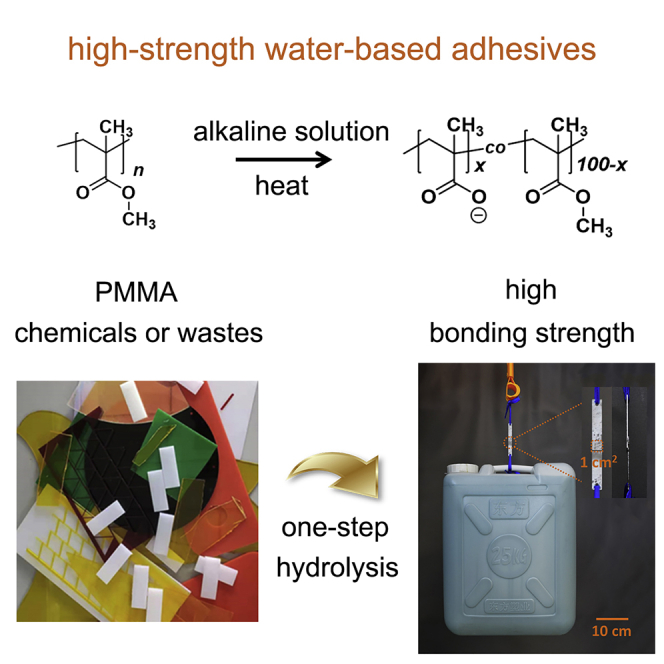

## Introduction

Nowadays, polymeric materials are playing an indispensable role in almost all aspects of our daily life and industrial fields.^1^^,^^2^^,^^3^^,^^4^ Each year, great amount of fossil raw is consumed to prepare plastic products.^5^ However, most of these plastic products are made into one-time products and become useless wastes when they are out of service or damaged, which as a result causes tremendous environmental pollution and resource waste.^6^ Therefore, the development of a facile and cost-effective way to achieve the reuse of these plastic wastes would be of great significance to the construction of a sustainable society.

Adhesives with the ability to conveniently bond diverse solid substrates together have been widely applied in various areas such as wood processing,^7^ construction,^8^ decoration,^9^ medical,^10^ automotive,^11^ electronics,^12^^,^^13^ and so forth. In recent decades, a variety of novel artificial adhesives have been prepared through biomimic the chemical composition of mussels,^14^^,^^15^^,^^16^^,^^17^^,^^18^ or the topological structure of biological tissues, such as gecko setae and octopus sucker,^12^^,^^19^^,^^20^ or a combination of the above two methods,^15^ to expand the application of adhesives in more specific fields. So far, most of the developed polymer adhesives were solvent-based ones because of their high bonding strength and fast solidification speed.^21^^,^^22^^,^^23^^,^^24^ However, the solvent-based adhesives would generally release massive volatile organic compounds (VOC) or toxic reagents during the process of use and drying, causing severe environment and health problems.^25^ Therefore, it is highly desirable to develop green and environment friendly polymer adhesives to substitute solvent-based adhesives. Recent years, more and more research interests were devoted to developing water-based adhesives due to their characteristics of no VOC release, safety, and environmental friendliness. The scope of water-based adhesive applications, however, is often severely limited by their poor bonding strength (usually in the range of kilopascals),^26^^,^^27^^,^^28^^,^^29^^,^^30^ which limited the application of these water-based adhesives in diverse areas. Therefore, the development of water-based adhesives with a high bonding strength comparable to those solvent-based adhesives would be a significant progress in the field of adhesives. Up to now, a few strategies had been developed to improve the bonding strength of water-based adhesives. Farinola and coworkers reported the preparation of all-water-based adhesives with high adhesive strength (2.5 MPa) via direct oxidative polymerization of dopamine in silk fibroin matrix followed by further crosslinking polydopamine by Fe^3+^ ions.^31^ Besides dopamine, some water-soluble organic molecules or polymers bearing multiple dopamine analogous reactive groups, such as catechol,^28^^,^^29^^,^^32^^,^^33^ pyrogallol,^34^ hydroxyl,^35^ and even the carboxyl^32^^,^^35^ groups, that were able to adhere to diverse surfaces based on supramolecular interactions were also adopted to prepare high-performance water-based adhesives. For example, Kim and coworkers prepared a strong adhesive that had a maximum adhesive strength ranging from ∼200 kPa on metal to 3.7 MPa on glass substrates by mixing poly(N-vinylpyrrolidone) and Tannic acid in aqueous solution.^33^ In our recent work, we demonstrated hydrogen bond-based poly(vinyl alcohol) and poly(acrylic acid) complexes prepared through one-step mixing of the two polymers in aqueous solution can produce super-high bonding strength for various kinds of substrates.^35^ Besides dopamine and its analogs, recently aqueous cellulose nanocrystal suspension that can self-assemble to form highly ordered hierarchical structure between solid surfaces had been demonstrated to have remarkable anisotropic adhesive between hard substrates (in-plane 7 MPa and out-of-plane≤0.08 MPa).^36^ Based on the current research on water-based adhesives, the supramolecular interactions played a critical role on preparing water-based adhesives. Because on the one hand, the adhesion force of water-based adhesives was mainly determined by the interfacial supramolecular interactions, including H-bonding, electronic interactions, coordination interactions, van der Waals interactions, and so forth, between the adhesives and the functional groups on different substrates.^31^^–^^36^ On the other hand, for water-based supramolecular adhesives, the cohesion force of the adhesives also depended on the supramolecular interactions that can hold the adhesives together.^33^^,^^35^ Although some progress had been made, the current methods for the preparation of water-based adhesives still face some main problems. Firstly, the preparation process of water-based adhesives generally involves harsh conditions with complicated and multi-step preparation procedures, which hinders the preparation of water-based adhesives at a large scale. Secondly, some raw materials used for synthesizing water-based adhesives are costly, which increases the production cost of these adhesives. Therefore, the development of a simple strategy utilizing low-cost chemicals or even polymer wastes as raw materials to achieve the large scaled preparation of water-based adhesives with high bonding strength on various substrates is highly desirable. Herein, polymethylmethacrylate (PMMA) chemicals or wastes were employed as raw materials for the facile preparation of multiple-substrate-applicable, high-strength, water-based adhesives through a simple one-step hydrolysis method. By controlling the hydrolysis degree of PMMA in an alkaline solution, water-based poly(methacrylic acid-co-methyl methacrylate) (denoted as P(MAA-MMA)) adhesives can be prepared, which exhibited high bonding strength toward different solid substrates, including metals, wood, and plastic substrates. In addition, the cohesion and adhesion force of the adhesives can be finely balanced by regulating the hydrolysis degree of PMMA to achieve the best bonding performance of the adhesives. Water-based P(MAA-MMA) adhesives exhibited a maximum bonding strength of 7.1 MPa to iron, 4.2 MPa to wood, and ∼1.5 MPa to plastic substrates. The high bonding strength of P(MAA-MMA) toward a broad range of substrates together with the simple preparation procedure utilizing PMMA wastes as raw materials not only makes P(MAA-MMA) an ideal water-based adhesive for large-scale industrial production and application but also contributes significantly to the construction of a sustainable society.

## Results and discussion

**Figure 1:**
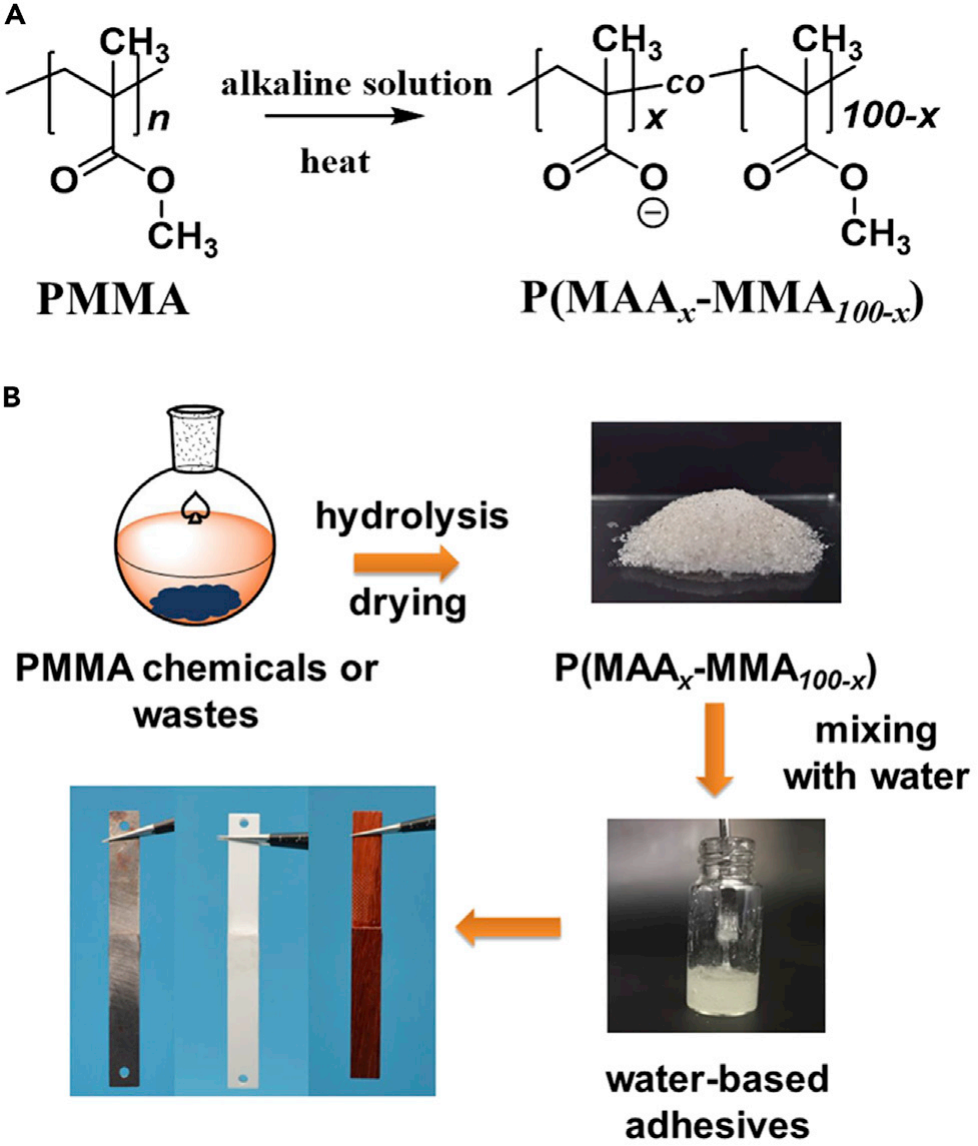
The preparation and the application of P(MAA_*x*_-MMA_*100-x*_) water-based adhesives (A) The preparation of P(MAA_*x*_-MMA*_100-x_*) copolymers by hydrolysis of PMMA in alkaline solution. (B) Schematic illustration of the preparation of water-based P(MAA_*x*_-MMA_*100-x*_) adhesive and the application of P(MAA_*x*_-MMA_*100-x*_) as high-strength adhesives toward different substrates.

PMMA chemicals or wastes were used as raw materials for preparing water-based P(MAA-MMA) adhesives by partially hydrolyzing the ester groups of PMMA to the carboxylate groups in an alkaline solution (Figure 1A). The hydrolysis degree of PMMA can be defined as the ratio of the ester groups that had been hydrolyzed into the carboxylate groups to all the ester groups of PMMA. The hydrolysis product of PMMA was denoted as P(MAA_*x*_-MMA_*100-x*_) with *x* being the hydrolysis degree of PMMA. PMMA chemicals were firstly used as the raw materials to study the preparation and bonding performance of water-based P(MAA_*x*_-MMA_*100-x*_) adhesives. Three P(MAA_*x*_-MMA_*100-x*_) copolymers with *x* value being ∼30%, ∼70%, and ∼90%, respectively, were prepared to investigate the influence of PMMA hydrolysis degree on the bonding strength of the adhesives. P(MAA_*x*_-MMA_*100-x*_) with *x* being ∼30% or ∼70% can be obtained through one-step hydrolysis of PMMA in KOH solution at 90°C for 48 h. To further increase the hydrolysis degree of PMMA, after hydrolyzing PMMA in KOH solution, the product was further hydrolyzed in 1 M NaOH solution at 120°C for 48 h to obtain the copolymers with hydrolysis degree of ∼90%. The successful preparation and exact compositions of the three P(MAA_*x*_-MMA_*100-x*_) polymers were firstly characterized by ^1^H NMR spectra. As shown in Figure S1, the characteristic peaks *c* at 3.61 ppm and peak *a* at ∼2.00 ppm on the ^1^H NMR spectrum of PMMA were assigned to the hydrogen atoms of the ester groups and methylene groups of PMMA, respectively. Compared with the ^1^H NMR spectrum of PMMA, the peak *c* at 3.61 ppm on the ^1^H NMR spectra of P(MAA_*x*_-MMA_*100-x*_) was obviously decreased, and the peak areas of peak *c* decreased with the increase in hydrolysis degree of PMMA, demonstrating the hydrolysis of the ester groups within PMMA. The hydrolysis degrees of PMMA can be calculated by the following formula:

$$hydrolysis\;degree\;x\;\%=\left(1-\frac{1}{3}A_{c}:\frac{1}{2}A_{a}\right)\times 100\;\%$$

where *A*_*c*_ and *A*_*a*_ were the peak areas of peak *c* and peak *a* in the ^1^H NMR spectra of P(MAA_*x*_-MMA_*100-x*_), respectively. And the results showed that the hydrolysis degrees were 37%, 66%, and 99%, respectively, for the three hydrolysis products of PMMA.

**Figure 2:**
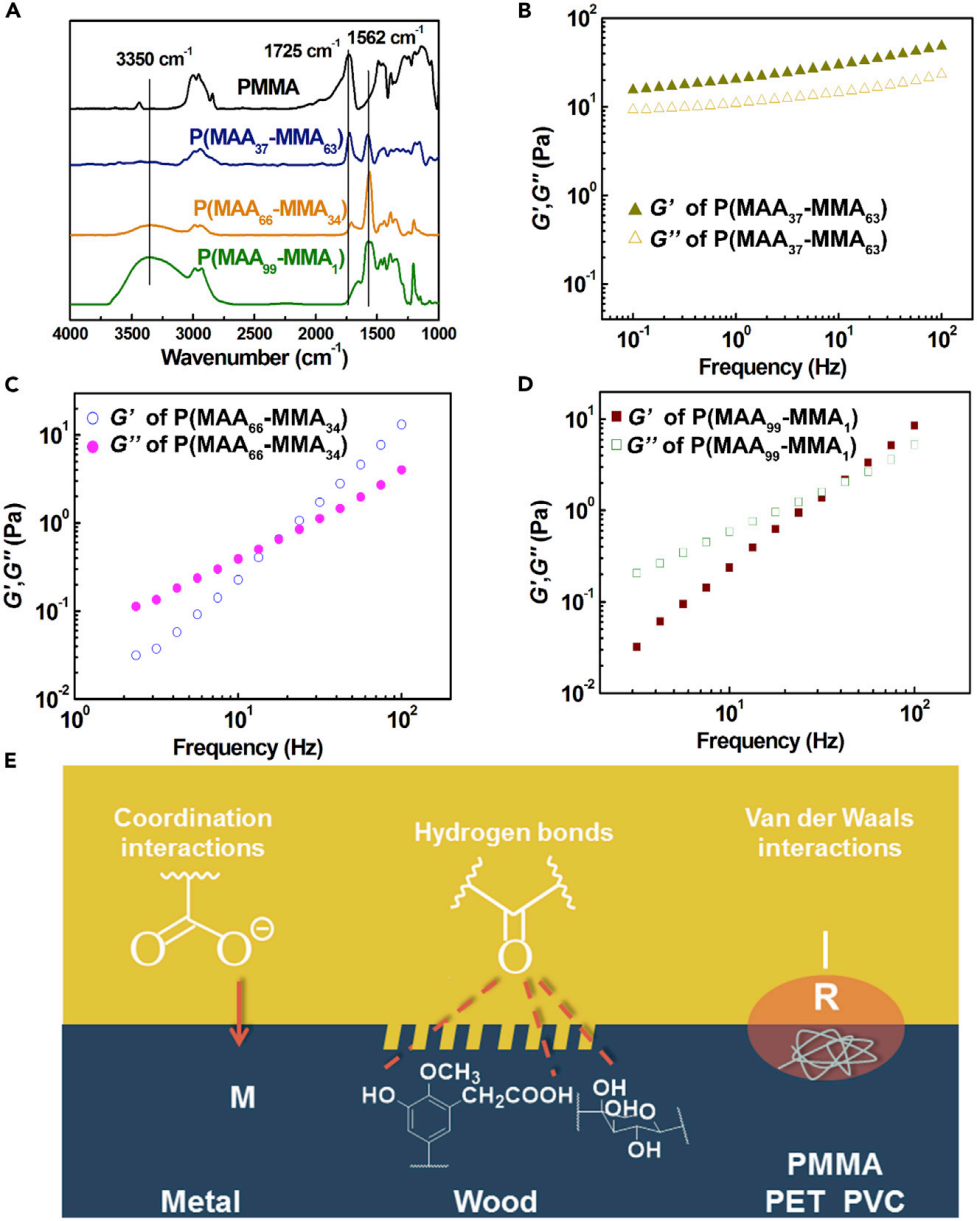
The FT-IR and rheological results of P(MAA_*x*_-MMA_*100-x*_), and the schematic illustration of the adhesion force of P(MAA_*x*_-MMA_*100-x*_) adhesives toward different substrates (A) The FT-IR spectra of PMMA, P(MAA_37_-MMA_63_), P(MAA_66_-MMA_34_), and P(MAA_99_-MMA_1_), respectively. (B–D) The storage modulus (*G′*) and loss modulus (*G″*) of P(MAA_37_-MMA_63_) (B), P(MAA_66_-MMA_34_) (C), and P(MAA_99_-MMA_1_) (D) as a function of shear frequency at a sweep frequency from 0.01 to 100 Hz, respectively. (E) Schematic illustration of the interfacial supramolecular interactions between P(MAA_*x*_-MMA_*100-x*_) copolymers and different substrates.

The Fourier transform infrared (FT-IR) spectra were also conducted to investigate the hydrolysis products of PMMA. As shown in Figure 2A, the characteristic peak at 1725 cm^−1^ on the FT-IR spectrum of PMMA chemical was attributed to the stretching vibration of the ester groups within PMMA. After the hydrolysis reaction, the characteristic peaks of the ester groups at 1725 cm^−1^ on the spectra of P(MAA_*x*_-MMA_*100-x*_) were obviously decreased. Meanwhile, two characteristic peaks that belonged to the stretching vibration of the carboxylate groups were appeared at 3350 and 1562 cm^−1^ on the spectra of P(MAA_*x*_-MMA_*100-x*_), respectively, indicating that part of the ester groups within PMMA have been successfully hydrolyzed into the carboxylate groups via hydrolysis reaction. With the increase in PMMA hydrolysis degree, the peak intensity of the carboxylate groups on the FT-IR spectra of P(MAA_*x*_-MMA_*100-x*_) at 3350 and 1562 cm^−1^ increased, whereas the peak intensity of the ester groups at ∼1725 cm^−1^ decreased, demonstrating the gradual increase in the contents of carboxylate groups in P(MAA_37_-MMA_63_), P(MAA_66_-MMA_34_), and P(MAA_99_-MMA_1_) polymers. The FT-IR results firmly proved the successful preparation of P(MAA_*x*_-MMA_*100-x*_) polymers with different hydrolysis degrees, and such results were in good agreement with ^1^H NMR results.

After drying and grinding, powdered P(MAA_*x*_-MMA_*100-x*_) copolymer is obtained, which is favorable for the storage and transportation. After mixing P(MAA_*x*_-MMA_*100-x*_) with water (the mass ratio between adhesive and water is 2:1), the adhesives become very viscous and can be utilized directly as water-based adhesives for various types of solid substrates (Figures 1B and S5). Within P(MAA-MMA) copolymers, there are both hydrophilic groups of carboxylate groups and hydrophobic groups of methyl and methacrylate groups, and these hydrophilic and hydrophobic groups played critical effects on the adhesion and cohesion force of the adhesives, respectively. The bonding performance of a water-based adhesive is depended on the synergy effect of both the cohesion force that can hold the adhesives together and the adhesion force between the adhesives and the substrates.^38^^,^^39^ As for P(MAA-MMA) copolymers, the cohesion force of the adhesives is provided mainly by the highly entanglement of the polymer chains driven though the hydrophobic-hydrophobic interactions of hydrophobic groups within the copolymers. The rheological measurements were performed to study the cohesion force of the P(MAA_*x*_-MMA_*100-x*_) adhesives with different hydrolysis degree. As shown in Figure 2B, the storage modulus (*G′*) of P(MAA_37_-MMA_63_) was always larger than the loss modulus (*G″*) over the entire frequency range from 10^−1^ to 10^2^ Hz, indicating the P(MAA_37_-MMA_63_) was always in a gel state due to the physical cross-linking of polymer chains by hydrophobic domains.^40^ Moreover, over the entire frequency range, both the *G′* and *G″* of P(MAA_37_-MMA_63_) polymer were much higher than that of the P(MAA_66_-MMA_34_) and P(MAA_99_-MMA_1_) in Figures 2C and 2D, respectively, indicating the cohesion force of P(MAA_37_-MMA_63_) adhesive was higher than that of the P(MAA_66_-MMA_34_) and P(MAA_99_-MMA_1_). By contrast, the *G′* and *G″* of both P(MAA_66_-MMA_34_) and P(MAA_99_-MMA_1_) were highly depended on the shear frequency with their *G″* greater than their *G′* in the low frequency range while *G′* greater than *G″* in the high frequency range. Such results suggested that both P(MAA_66_-MMA_34_) and P(MAA_99_-MMA_1_) remained in solution state at low shear frequency. Moreover, with the increase in shear frequency, the P(MAA_66_-MMA_34_) firstly reached to the gel state when the shear frequency was over 18 rad/s, while the sol-gel transition of P(MAA_99_-MMA_1_) solution occurred only after the shear frequency was larger than 45 rad/s, suggesting that it took a longer time for P(MAA_66_-MMA_34_) to achieve the stress relaxation than that of the P(MAA_99_-MMA_1_). Therefore, the cohesion force of P(MAA_66_-MMA_34_) was higher than that of the P(MAA_99_-MMA_1_). The rheological results demonstrated that the cohesion force of P(MAA_*x*_-MMA_*100-x*_) adhesives decreased with the increasing in the hydrolysis degree of PMMA.

Besides the cohesion force, the interfacial adhesion force between the adhesives and the substrates was also critically important for determining the bonding strength of the adhesives. As illustrated in Figure 2E, the carboxylate groups of P(MAA_*x*_-MMA_*100-x*_) adhesives can establish the coordination bond and hydrogen bond interactions with the hydrophilic substrates of metals and wood, respectively. While, the van der Waals interactions played a critical role on the adhesion of P(MAA_*x*_-MMA_*100-x*_) adhesives toward plastic substrates, such as PMMA, polyethylene terephthalate (PET), and polyvinyl chloride (PVC). For metal and wood surfaces, the adhesion force should be proportional to the content of carboxylate groups within the adhesives. Therefore, with the increase in hydrolysis degree, the adhesion force of the P(MAA_*x*_-MMA_*100-x*_) adhesives increases while the cohesion force decreases. Considering the balance between cohesion and adhesion of P(MAA_*x*_-MMA_*100-x*_) adhesives, it can be concluded that the optimized balance between the hydrophilic and hydrophobic segments within the copolymers is the key factor to achieve the maximized bonding strength of the adhesives.^35^^,^^38^

**Figure 3:**
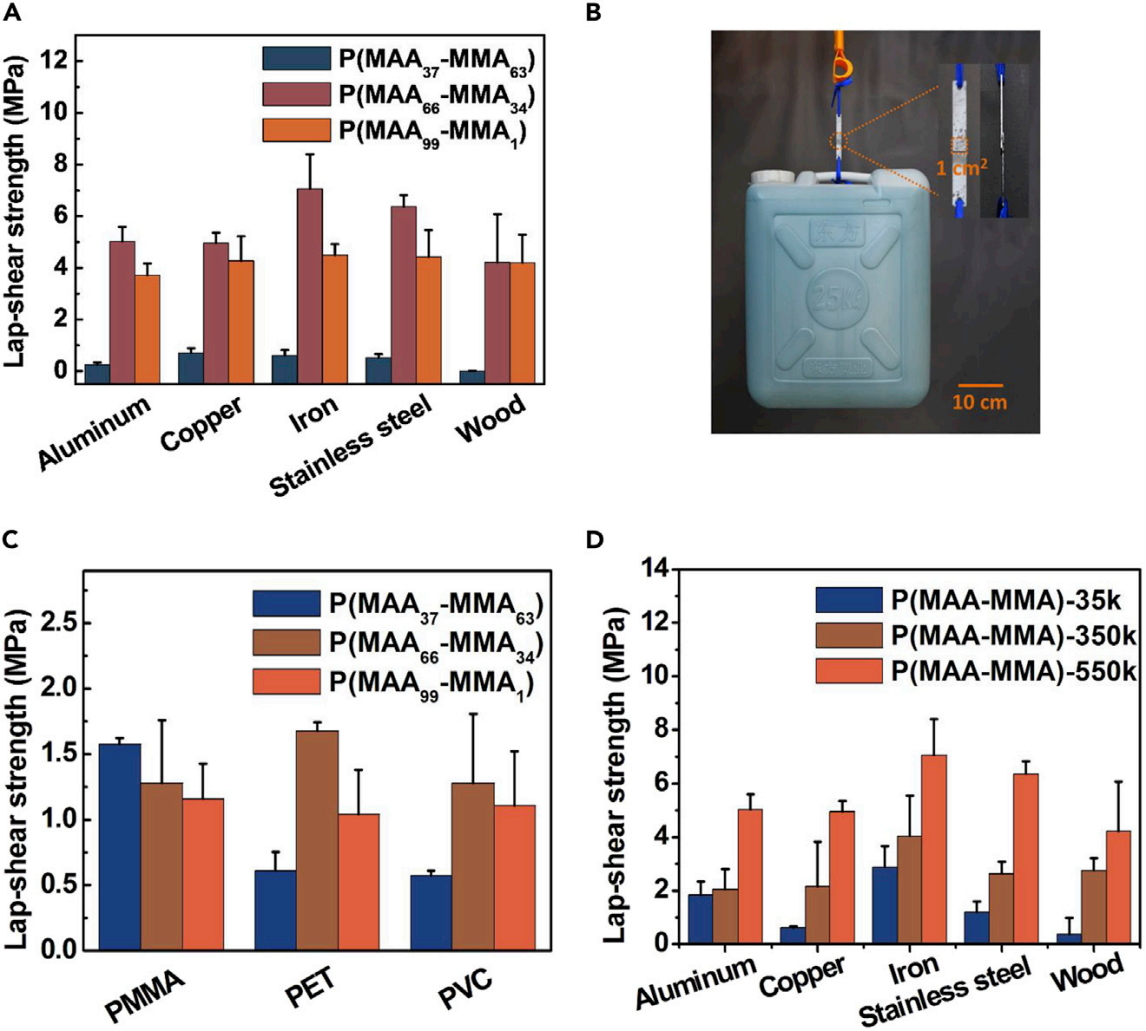
The bonding strength of P(MAA_*x*_-MMA_*100-x*_) water-based adhesives (A) The bonding strength of P(MAA_37_-MMA_63_), P(MAA_66_-MMA_34_), and P(MAA_99_-MMA_1_) on metal and wood substrates. (B) Digital photo of two pieces of iron sheets adhered together by water-based P(MAA_66_-MMA_34_) adhesive with a lap area of 1 × 1 cm^2^ lifting a weight of 25 kg. (C) The bonding strength of P(MAA_37_-MMA_63_), P(MAA_66_-MMA_34_), and P(MAA_99_-MMA_1_) toward plastic substrates, including PMMA, PET, and PVC. (D) The influence of PMMA molecular weight on the lap-shear strength of the adhesives to metal and wood substrates.

The bonding strength of water-based P(MAA_*x*_-MMA_*100-x*_) adhesives to a broad variety of substrates, including metals, wood, and plastics, with different surface energy was evaluated through the lap-shear tests after the samples were completely dried at ambient environment. As shown in Figure 3A, for each kind of metal substrates, including aluminum, copper, iron, and stainless steel, the bonding strength of the adhesives followed the subsequence that P(MAA_66_-MMA_34_) > P(MAA_99_-MMA_1_) > P(MAA_37_-MMA_63_). The P(MAA_66_-MMA_34_) adhesives exhibited the best bonding performance toward all the metal substrates with the average bonding strength larger than 5 MPa. The bonding strength of P(MAA_66_-MMA_34_) to metals is almost the strongest when compared with that of current reported water-based adhesives,^26^^–^^30^ and the bonding strength of water-based P(MAA_66_-MMA_34_) adhesives is comparable or even superior to that of the solvent-based 3M epoxy glue (Table S1). The P(MAA_66_-MMA_34_) adhesives had a maximum bonding strength because it had the optimum balance between the cohesion and adhesion within this adhesive. Further increase in the hydrolysis degree of the adhesives can lead P(MAA_99_-MMA_1_) to have a higher adhesion force but lower cohesion force; on the contrary, decreasing the hydrolysis degree of the adhesives can result in P(MAA_37_-MMA_63_) possess a higher cohesion force but lower adhesion force. The mismatch between cohesion and adhesion resulted in the detachment of adhesives at the weak place first when the adhesives were subjected to external force.^21^^,^^39^ Therefore, the bonding strength of P(MAA_99_-MMA_1_) and P(MAA_37_-MMA_63_) was not as high as that of the P(MAA_66_-MMA_34_). Besides the relatively low adhesion force, P(MAA_37_-MMA_63_) was in a gel state at room temperature, which made it difficult to contact adequately with the substrates; therefore, the P(MAA_37_-MMA_63_) had the lowest bonding strength among these three adhesives. In addition, it is worth noting that among these metal substrates, P(MAA_66_-MMA_34_) exhibited the maximum adhesion toward iron with its bonding strength as high as 7.1 ± 1.3 MPa because the coordination interaction between the carboxylate groups and the iron atom is the strongest when compared with other metals.^41^ As shown in Figure 3B, two pieces of iron sheets adhered together by water-based P(MAA_66_-MMA_34_) adhesive with a lap area of 1 × 1 cm^2^ can easily lift up a 25 kg weight. Besides metal substrates, P(MAA_66_-MMA_34_) and P(MAA_99_-MMA_1_) also exhibited excellent adhesive performance to wood based on the hydrogen bond interactions between the adhesives and cellulose or lignin on wood (Figure 2E). P(MAA_66_-MMA_34_) and P(MAA_99_-MMA_1_) had similar bonding strength of ∼4.2 MPa toward wood probably because the rough and porous surface structure of the wood for one hand enhanced the adhesion force between adhesive and wood by increasing the adhesion area, and for the other hand, decreased the cohesion force of the adhesives by partially interfering with the chain entanglements of the adhesives.

Water-based P(MAA_*x*_-MMA_*100-x*_) adhesives also exhibited satisfactory bonding behavior to commonly used plastics materials, including PMMA, PET, and PVC (Figure 3C). For these plastics substrates, the cohesion force of the adhesives was much higher than that of the interfacial adhesion force (van der Waals force) between the adhesives and plastic substrates (Figure 2E). Therefore, the bonding strength of adhesives was mainly determined by the van der Waals force between the adhesives and plastic substrates because when the adhesive is subjected to an external force, the adhesive will first break at the position where the force is weak. Accordingly, the bonding strength of the adhesives toward plastic substrates was not as high as that toward metal and wood substrates. For PET and PVC substrates, P(MAA_66_-MMA_34_) exhibited the highest lap-shear strength of 1.7 ± 0.1 and 1.3 ± 0.5 MPa for PET and PVC, respectively. For PMMA substrates, the bonding strength of the three adhesives is comparable with the best lap-shear strength of 1.6 ± 0.1 MPa. For all these plastics substrates, the best bonding strength of water-based P(MAA_*x*_-MMA_*100-x*_) adhesives was larger than 1.3 MPa, demonstrating their excellent bonding performance for plastic materials.^21^^,^^39^

Based on the above experiments, it can be concluded that water-based P(MAA_66_-MMA_34_) adhesives possessed almost the best bonding performance to metals, wood, and most of the plastic materials; therefore, the same hydrolysis conditions for preparing P(MAA_66_-MMA_34_) copolymers were applied in the following experiments. The cross-sectional SEM images of P(MAA_66_-MMA_34_) adhesives used for bonding two iron substrates were shown in Figure S6. SEM results show that there are spherical particles embedded within P(MAA_66_-MMA_34_) matrix. The spherical particles are formed probably through the highly entanglement of P(MAA_66_-MMA_34_) chains driven by hydrophobic-hydrophobic interactions when the copolymers were mixed with water, which further proved the formation of cohesion force within P(MAA-MMA) adhesives. The differential scanning calorimetry results show that P(MAA_66_-MMA_34_) has a *T*_g_ of 110°C (Figure S7). The thermal gravimetric analysis results show that P(MAA_66_-MMA_34_) adhesives have a water content of ∼1% (Figure S8). The influence of PMMA molecular weight on the lap-shear strength of P(MAA_66_-MMA_34_) adhesives to hydrophilic substrates was illustrated in Figure 3D. For metal and wood substrates, the bondings strength of the adhesives increased with the molecular weight of PMMA because the higher molecular weight of PMMA can lead to more entanglement of polymer chains, resulting in larger cohesion of the adhesives (Figure 3D). Therefore, PMMA with the highest molecular weight of 550 kDa showed best bonding behaviors to all the metal and wood substrates. Water-based P(MAA-MMA) adhesives also exhibited excellent stability at room temperature (25°C) with relative humidity (RH) of 50%. After storage in the ambient environment (25°C, RH ∼50%) for up to 10 months, the lap-shear strength of P(MAA_66_-MMA_34_) to iron sheets can still be maintained at 6.0 MPa, demonstrating satisfactory stability of the adhesives in the ambient environment.

**Figure 4:**
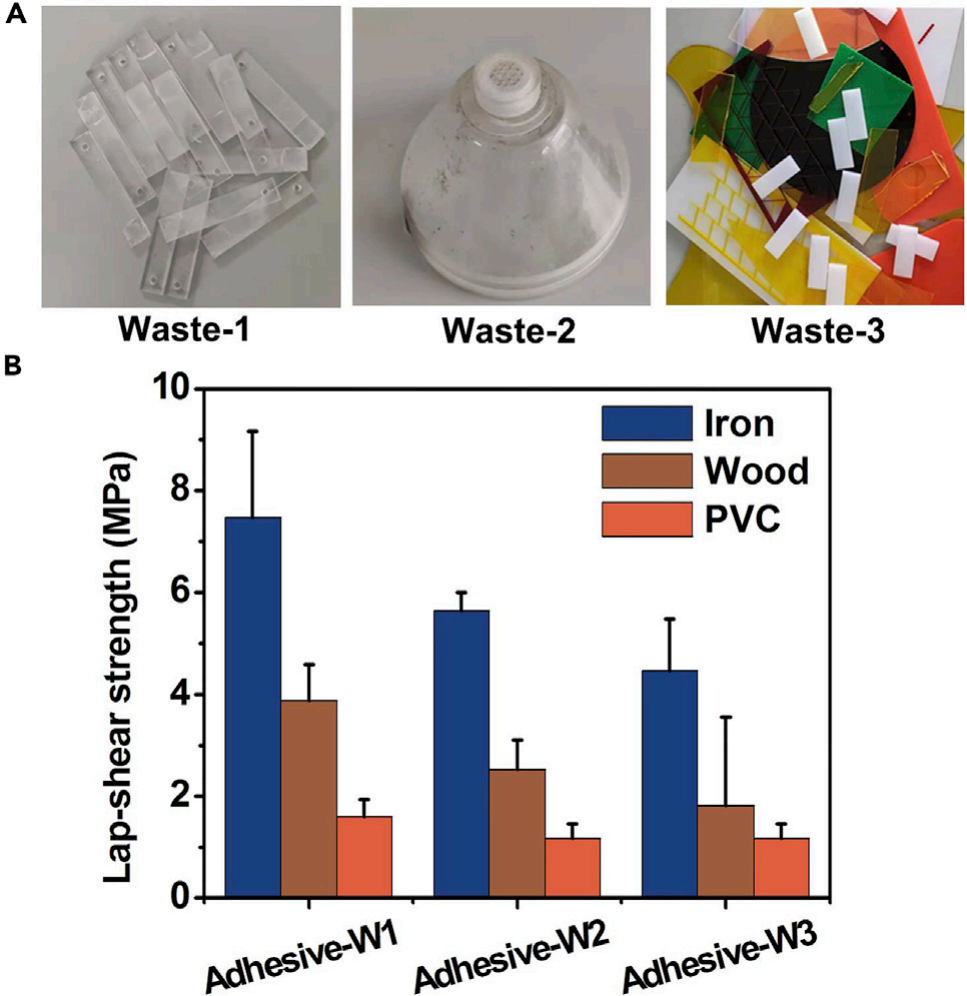
The preparation of water-based P(MAA-MMA) adhesives using PMMA wastes (A) Digital photos of the three PMMA wastes used for preparing water-based P(MAA-MMA) adhesives. (B) The lap-shear strength of Adhesive-W1, Adhesive-W2, and Adhesive-W3 prepared from PMMA wastes to iron, wood, and PVC.

It is noteworthy that PMMA wastes could be used as the raw materials for preparing water-based P(MAA-MMA) adhesives with high bonding strength. As shown in Figure 4A, three PMMA wastes (denoted as Waste-1, Waste-2, and Waste-3, respectively) that were randomly obtained from the waste recycle stations were taken as typical examples to show the preparation of adhesives from PMMA wastes. The ^1^H NMR spectra of Waste-1, Waste-2, and Waste-3 were shown in Figures S3A–S3C, respectively. Among these PMMA wastes, Waste-3 was the mixture of multiple PMMA wastes. The three PMMA wastes were hydrolyzed respectively according to the same method for preparing P(MAA_66_-MMA_34_) copolymers from PMMA chemical for 3 days, and their hydrolysis products were named as Adhesive-W1, Adhesive-W2, and Adhesive-W3, respectively. The ^1^H NMR spectra of Adhesive-W1, Adhesive-W2, and Adhesive-W3 were shown in Figures S3D–S3F, respectively. As shown in Figure 4B, toward different types of substrates, all the adhesives prepared from PMMA wastes exhibited a similar bonding performance to that of the P(MAA_66_-MMA_34_) copolymers prepared by hydrolyzing PMMA chemicals with the adhesives having the highest bonding strength to iron, second to wood, and the lowest to PVC. Among these three adhesives prepared from PMMA wastes, Adhesive-W1 showed the best bonding performance with the lap-shear strength of 7.5 ± 1.7, 3.9 ± 0.7, and 1.6 ± 3.4 MPa to iron, wood, and PVC, respectively, which were highly comparable to that of the P(MAA_66_-MMA_34_) adhesives prepared from PMMA chemicals. For the hydrolysis products of PMMA mixture, the Adhesive-W3 also exhibited superior adhesive properties to different substrates with the lap-shear strength of 4.5 ± 1.0, 1.8 ± 1.7, and 1.2 ± 0.3 MPa to iron, wood, and PVC, respectively, demonstrating that it is a universally applicable method for preparing high-strength water-based adhesives to different types of substrates from the PMMA wastes.

### Conclusions

In summary, water-based adhesives with high bonding strength to a broad variety of substrates were facilely prepared through one-step hydrolysis of PMMA chemicals or wastes in a strong alkaline environment. By tuning the hydrolysis conditions of PMMA, P(MAA_*x*_-MMA_*100-x*_) copolymers with controllable hydrolysis degree can be obtained, which can be utilized as water-based adhesives to multiple types of substrates, including metals, wood, and plastic. The hydrophobic segments within P(MAA_*x*_-MMA_*100-x*_) copolymers can drive the polymers to form highly entangled structure, which worked as the cohesion force of the adhesives. Meanwhile, the various supramolecular interactions between the carboxylate groups of P(MAA_*x*_-MMA_*100-x*_) copolymers and the substrates played a critical role on the adhesion force of the adhesives toward diverse surfaces. The cohesion force and the adhesion force of water-based P(MAA_*x*_-MMA_*100-x*_) adhesives can be finely balanced by controlling the hydrolysis degree of PMMA. The results showed that the P(MAA_66_-MMA_34_) had the optimum balance between the cohesion and adhesion, and had a maximum bonding strength up to 7.1 MPa to iron, 6.4 MPa to stainless steel, 4.2 MPa to wood, and ∼1.5 MPa to plastic substrates. The P(MAA_66_-MMA_34_) adhesives have a world-record bonding strength to metal substrates when compared with that of current reported water-based adhesives, and the bonding strength is comparable or even superior to that of the solvent-based 3M epoxy glue. More importantly, the PMMA wastes can be hydrolyzed according to the same method for preparing P(MAA_66_-MMA_34_) adhesives from PMMA chemicals, and the resulted water-based adhesives also exhibited a high bonding strength toward various substrates. Our method for preparing water-based adhesives is super simple and readily to be scaled up. The as-developed water-based adhesives possessed excellent bonding performance with long-term stability toward various kinds of substrates. More importantly, the raw materials used for preparing these high-strength water-based adhesives can be PMMA wastes, which not only can effectively decrease the production costs of the adhesives but also can greatly contribute to the construction of a sustainable society. It is believed that the water-based P(MAA-MMA) adhesives with all above merits will show great promise for the wide applications in daily life and industries.

### Limitations of the study

Water-based P(MAA_66_-MMA_34_) adhesives possess high bonding strength toward various substrates with excellent stability in the ambient environment; however, the bonding strength and the stability of the adhesive in water are not so satisfactory as that in air, which limit the application of P(MAA_66_-MMA_34_) adhesives in water.

## STAR★Methods

### Key resources table

**Table:** 

REAGENT or RESOURCE	SOURCE	IDENTIFIER
PMMA(35 kDa)	Alfa Aesar	CAS no. 9011-14-7
PMMA(350 kDa)	Acros	CAS no. 9011-14-7
PMMA(550 kDa)	Sigma-Aldrich	CAS no.9011-14-7
Methanol	Tianjin Xinbote Chemical Co., Ltd.	CAS no. 67-56-1
1,4-dioxane	Tianjin Xinbote Chemical Co., Ltd.	CAS no. 123-91-1
NaOH	Tianjin Guangfu Technology Development Co. Ltd.	CAS no. 1310-73-2
KOH	Sinopharm Chemical Reagent Co., Ltd.	CAS no. 1310-58-3

### Resource availability

#### Lead contact

Further information and requests for resources and reagents should be directed to and will be fulfilled by the lead contact, Ling Zhang (zhanglingchem@jlu.edu.cn).

#### Material availability

This study did not generate new unique reagents.

### Method details

#### Synthesis of P(MAA-MMA) adhesives from PMMA chemicals

Water-based P(MAA_*x*_-MMA_*100-x*_) adhesives with the hydrolysis degree being *x* % were synthesized through the hydrolysis method.^21^^,^^37^

#### Synthesis of P(MAA_66_-MMA_34_)

The P(MAA_66_-MMA_34_) was taken as a typical example to show the synthesis procedure of P(MAA_*x*_-MMA_*100-x*_) copolymers. PMMA (*M*_*w*_ ≈ 550 kDa, 10 g) was firstly dissolved in 1,4-dioxane (100 mL) at 90 °C. After the system was cooled to 60 °C, KOH (11.2 g in 30 mL methanol) was added in the PMMA solution. The hydrolysis reaction was allowed to proceed at 90 °C for 48 h, after which the product was precipitated from the solution. The as-prepared product was allowed to dialyze in deionized water for three days and then dried at 80 °C. ^1^H NMR (500 MHz, D_2_O) of P(MAA_66_-MMA_34_) was shown in Figure S1B.

#### Synthesis of P(MAA_37_-MMA_63_)

The P(MAA_37_-MMA_63_) was synthesized and purified by a similar procedure as that of the P(MAA_66_-MMA_34_), except that less KOH (8.4 g) was required for the hydrolysis of PMMA. ^1^H NMR (500 MHz, MeOD) of P(MAA_37_-MMA_63_) was shown in Figure S1C.

#### Synthesis of P(MAA_99_-MMA_1_)

The P(MAA_99_-MMA_1_) was synthesized by two-step hydrolysis of PMMA. Firstly, PMMA (*M*_w_ ≈ 550 kDa, 10 g) was dissolved in 1,4-dioxane (100 mL) at 90 °C, then KOH (11.2 g in 30 mL methanol) was added after the temperature of the PMMA solution was cooled to 60 °C. The PMMA was allowed to hydrolyzed in KOH at 90 °C for 48 h, and the product was precipitated from the solution. After pouring out the supernatant, NaOH aqueous solution (1 M, 100 mL) was added to the reaction system and the reaction was refluxed at 120 °C for 48 h to further improve the hydrolysis degree of PMMA. After the hydrolysis reaction, the resultant solution was cooled to the room temperature, and subsequently dialyzed for three days in deionized water for purity. The P(MAA_99_-MMA_1_) was finally obtained after drying at 80 °C. ^1^H NMR (500 MHz, D_2_O) of the P(MAA_99_-MMA_1_) was shown in Figure S1D.

#### Hydrolysis of PMMA chemicals with different molecular weight

The hydrolysis procedure of PMMA with molecular weight of 35 and 350 kDa was similar to that of preparing P(MAA_66_-MMA_34_) from PMMA (*M*_w_ ≈ 550 kDa) except using PMMA with molecular weight of *M*_w_ ≈ 35 kDa or 350 kDa to substitute for PMMA (*M*_w_ ≈ 550 kDa) as the starting reactant, respectively. And the hydrolysis products were denoted as P(MAA-MMA)-35k and P(MAA-MMA)-350k, respectively. The ^1^H NMR (500 MHz, D_2_O) of the P(MAA-MMA)-35k and P(MAA-MMA)-350k were shown in Figure S2.

#### Synthesis of P(MAA-MMA) adhesives from PMMA wastes

PMMA wastes were obtained randomly from the waste recycling plant. Three kinds of PMMA wastes (denoted as PMMA-W1, PMMA-W2 and PMMA-W3, respectively) were chosen as typical examples to show the general preparation method of water-based adhesives from PMMA wastes. All the PMMA wastes were hydrolyzed by one-step hydrolysis method according to the similar procedures to that of preparing P(MAA_66_-MMA_34_) from PMMA chemicals. Specifically, after thoroughly cleaned with water, PMMA wastes (10 g) were firstly dissolved in 1,4-dioxane (100 mL) at 90 °C. Then KOH (11.2 g in 30 mL methanol) was added to the PMMA solution after the temperature of the system was cooled to 60 °C and the hydrolysis reaction was allowed to proceed at 90 °C for 72 h. After the reaction, the product was precipitated from the solution, and dialyzed for three days in deionized water and further dried at 80 °C to obtain the purified adhesives. The hydrolysis products of PMMA-W1, PMMA-W2, and PMMA-W3 were named as Adhesive-W1, Adhesive-W2, and Adhesive-W3, respectively. ^1^H NMR (500 MHz, D_2_O) of Adhesive-W1, Adhesive-W2, and Adhesive-W3 were shown in Figures S3D–S3F, respectively.

#### Lap-shear test

When 1 g P(MAA-MMA) was mixed with 0.5 mL water, only P(MAA_37_-MMA_63_) with a molecular weight of 550 kDa was in a gel state and cannot be dissolved in water, other adhesives, including P(MAA_66_-MMA_34_) and P(MAA_99_-MMA_1_) with different molecular weight, and P(MAA_37_-MMA_63_) with a molecular weight of 35 kDa, can all be completely dissolved in water. After mixing with water, the powdered P(MAA-MMA) can turn into a viscous liquid like glue, and can be used directly as adhesives for solid substrates. The bonding process was shown in Figure S4, the adhesives were evenly applied to one substrate with a lap area of 1 × 1 cm^2^. Then the other substrate was completely covered on the adhesives. After that, a pressure of ∼10 kPa was applied to the adhesive area of the substrates. After being completely dried at ambient conditions, the bonding strength of the adhesives was measured through lap-shear tests using the universal testing machine (410R250 Tension Instrument) at an ambient condition under a stretching speed of 50 mm/min. Except wood, all the other substrates were polished with 360-grit sandpaper for 30 s before applying the adhesives. For each sample, 5 times of lap-shear tests were conducted to obtain the average adhesion strength of the adhesives. And the error bars showed the relative standard deviation of the bonding strength of the adhesives.

#### Instruments and characterizations

The Fourier transform infrared (FT-IR) spectra were tested by a Bruker VERTEX 80 V FT-IR spectrometer. ^1^H NMR spectra were carried out on a 500 MHz Bruker AVANCE III spectrometer. Rheological measurements were performed at 25 °C on a TA Instrument HR-2 rheometer with 40 mm parallel stainless-steel plates. The frequency sweeps were conducted at a constant shear strain of 2% by varying angular frequency from 0.1 to 100 rad/s. The lap-shear strengths were measured by 410R250 Tension Instrument (TestResources Inc., USA) at a stretching speed of 50 mm/min. The digital images were captured by a Canon PowerShot SX40 HS camera. The thermal gravimetric analysis (TGA) measurements were tested on a Q500 thermogravimetric analyzer (TA Instruments) under a nitrogen atmosphere at a heating rate of 10 °C/min. Scanning electron microscopy (SEM) was conducted under vacuum using a Hitachi SU8020 SEM (Tokyo, Japan). Differential scanning calorimetry (DSC) measurements were performed on a TA Instruments Q200 differential scanning calorimeter under a nitrogen flow of 50 mL/min.

## Data Availability

• Data reported in this article will be shared by the lead contact on request. • There is no dataset or code associated with this work.
