# Supplementary material for: Supramolecular interaction enabled preparation of high-strength water-based adhesives from polymethylmethacrylate wastes
Source: iScience. 2023 Jan 23;26(2):106022. doi: 10.1016/j.isci.2023.106022 (PMC9932134; doi:10.1016/j.isci.2023.106022)
Supplement: Document S1. Figures S1–S8 and Table S1 [file mmc1.pdf]

iScience, Volume *26*

Supplemental information

Supramolecular interaction enabled preparation of high-strength water-based adhesives from polymethylmethacrylate wastes

Jing Kang, Xiang Li, Yunlu Zhou, and Ling Zhang

Supporting Information

Supramolecular-interaction enabled preparation of high-strength water-based adhesives from polymethylmethacrylate wastes

Jing Kang[1], Xiang Li[1],Yunlu Zhou[1], and Ling Zhang[1,2] *

[1] State Key Laboratory of Supramolecular Structure and Materials, College of Chemistry, Jilin University, Changchun 130012, P. R. China.

[2] **Ling Zhang** is the Lead Contact

[*]Correspondence: zhanglingchem@jlu.edu.cn.

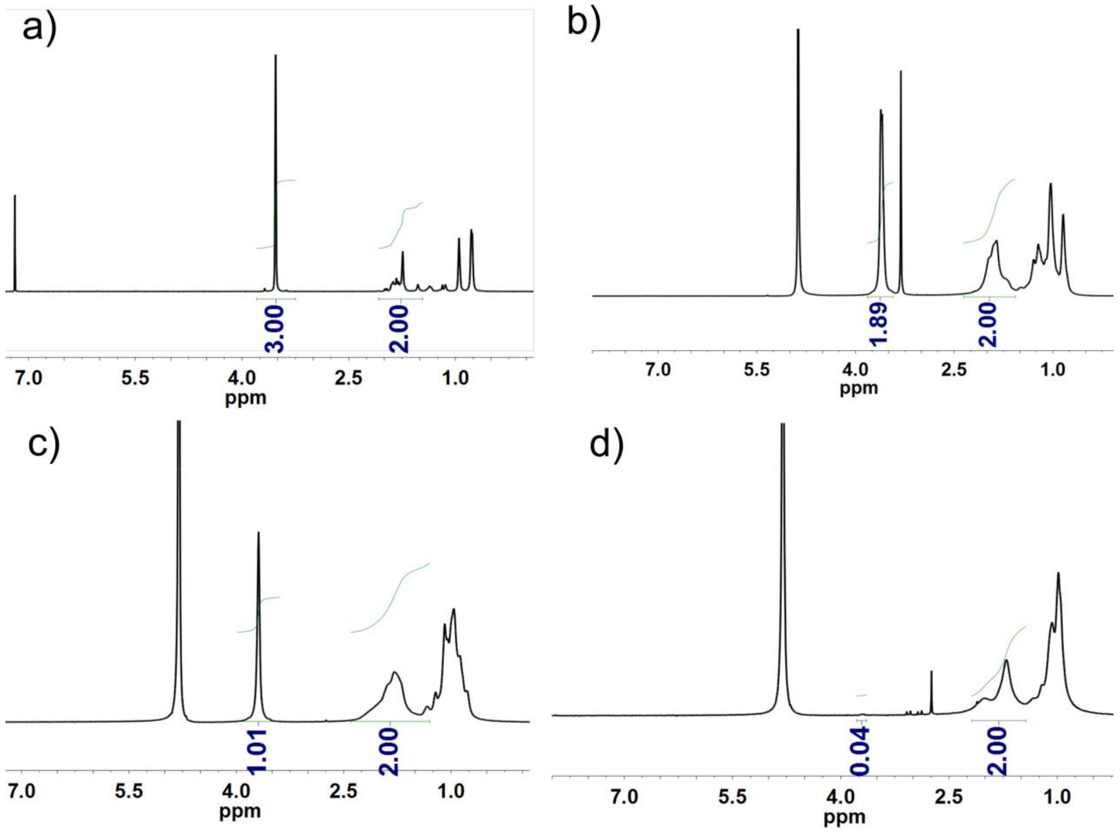

Fig. S1. The ^{1}H NMR spectra of PMMA and P(MAA$_x$-MMA$_{100-x}$), Related to STAR Methods.

(a) The ^{1}H NMR spectrum of PMMA. (b-d) The ^{1}H NMR spectra of P(MAA$_{37}$-MMA$_{63}$) (b), P(MAA$_{66}$-MMA$_{34}$) (c), and P(MAA$_{99}$-MMA$_1$) (d), respectively.

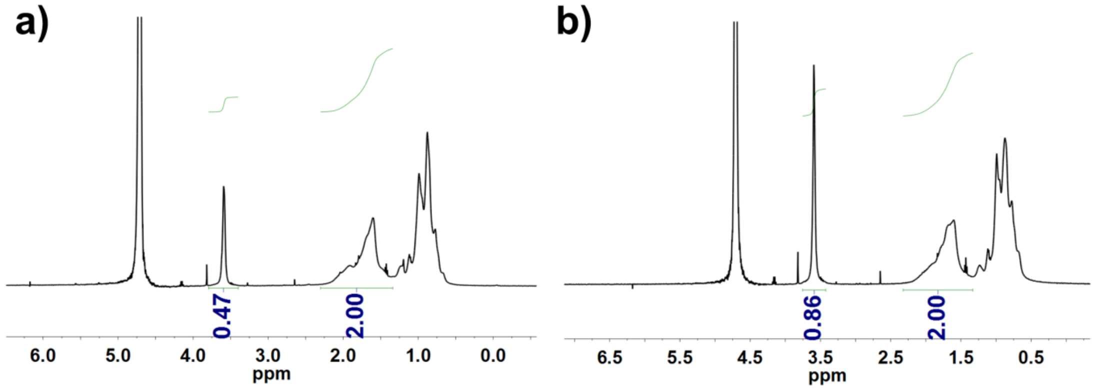

Fig. S2. The ^{1}H NMR spectra of P(MAA$_{x}$-MMA$_{100\text{-}x}$) prepared from PMMA chemicals with different molecular weight, Related to STAR Methods.

(a) The ^{1}H NMR spectrum of P(MAA-MMA)-35k, (b) the ^{1}H NMR spectrum of P(MAA-MMA)-350k.

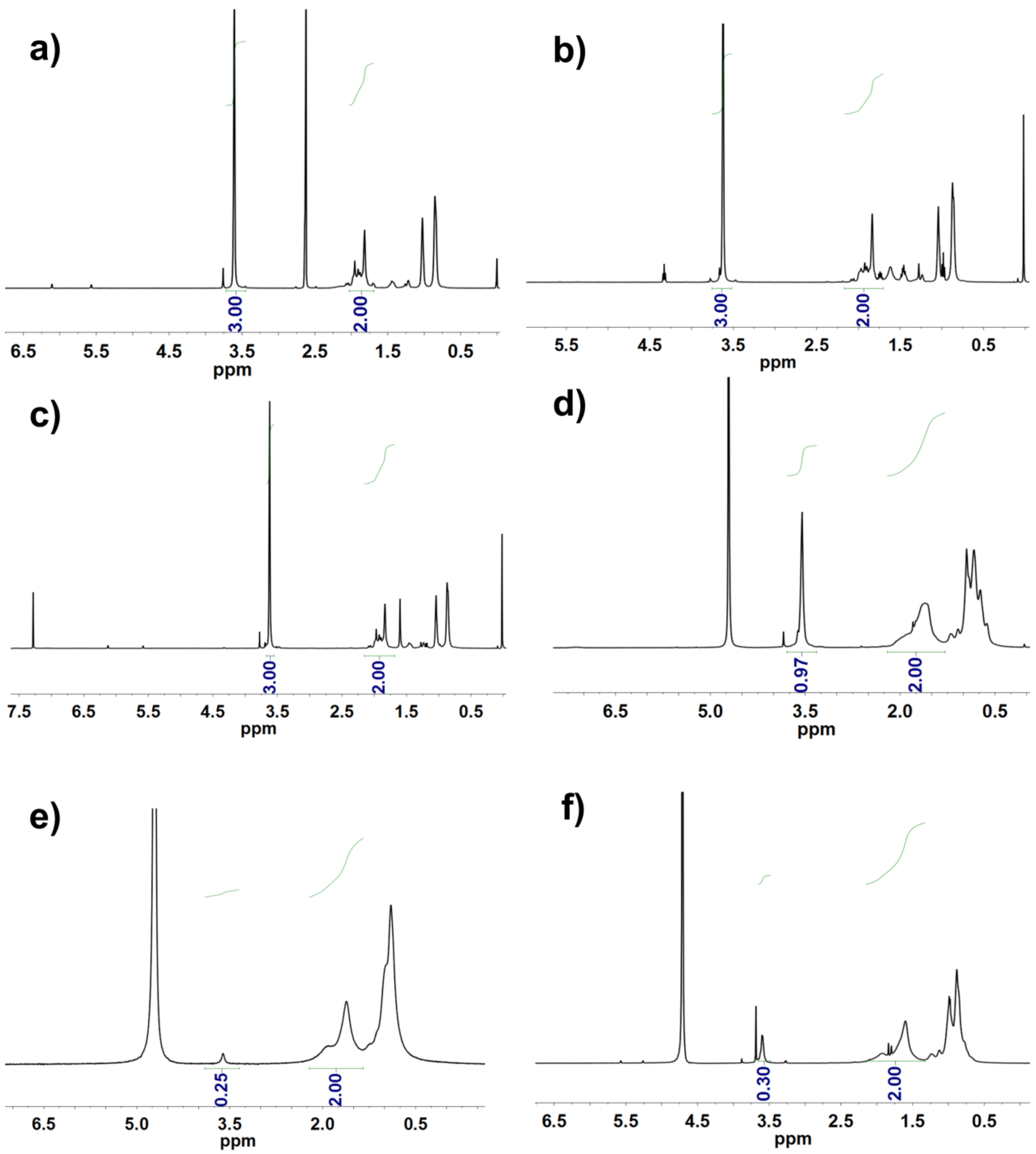

Fig. S3. The ^{1}H NMR spectra of PMMA wastes and their corresponded hydrolysis productions, Related to STAR Methods.

(a-c) The ^{1}H NMR spectra of Waste-1 (a), Waste-2 (b), and Waste-3 (c), respectively. (d-f) The ^{1}H NMR spectra of Adhesive-W1 (d), Adhesive-W2 (e), and Adhesive-W3 (f), respectively.

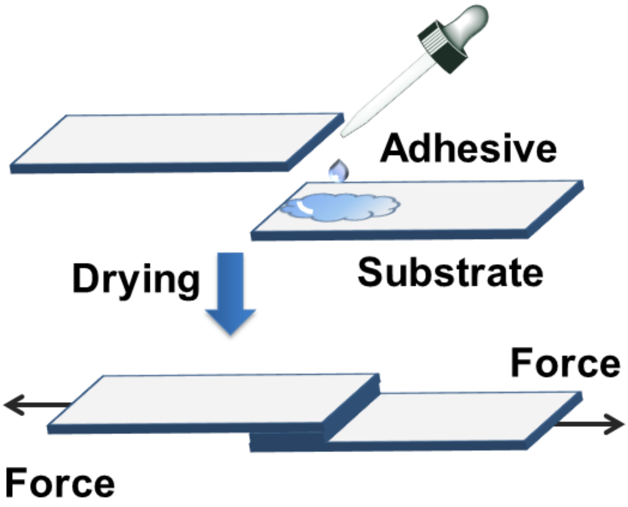

Fig. S4. Schematic illustration of the bonding process and lap-shear test, Related to STAR Methods.

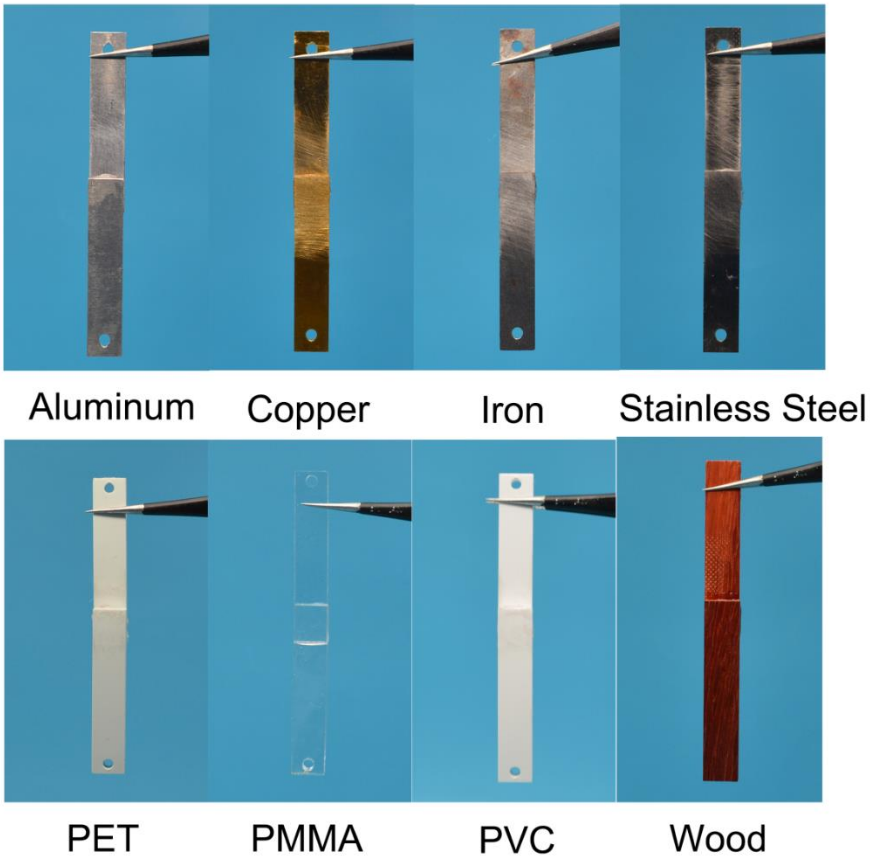

Fig. S5. Digital photos of different substrates adhered together by water-based P(MAA_{66}-MMA_{34}) adhesive (lap area 1 × 1 cm^2), respectively, Related to STAR Methods.

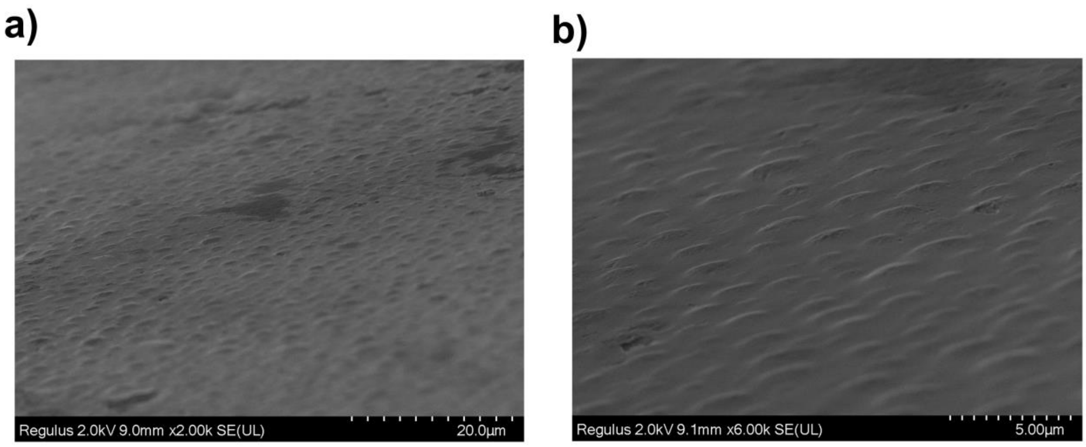

Fig. S6. The cross-sectional SEM images of P(MAA_{66}-MMA_{34}) adhesives, Related to STAR Methods.

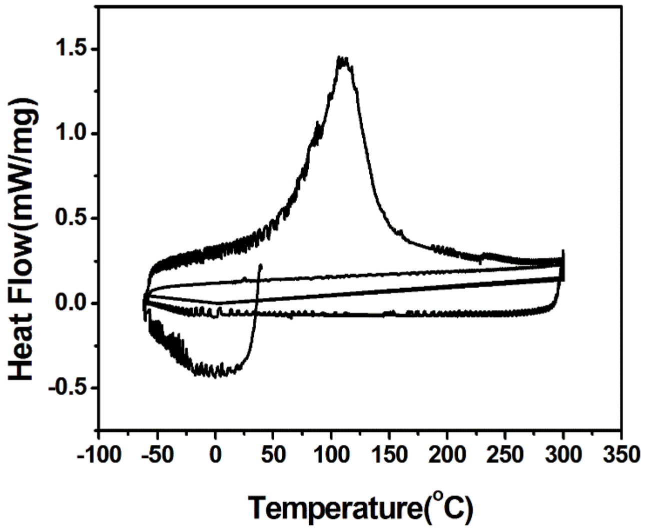

Fig. S7. The DSC curve of P(MAA_{66}-MMA_{34}) adhesive, Related to STAR Methods.

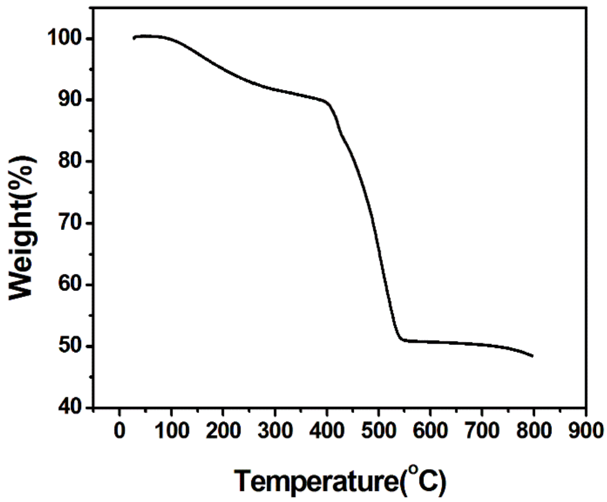

Fig. S8. The TGA curve of P(MAA_{66}-MMA_{34}) adhesive, Related to STAR Methods.

Table S1. Lap-shear strength of 3M epoxy adhesive (DP100 Clear) on different substrates, Related to STAR Methods.

Substrates	Bonding Strength (MPa)
Aluminum	6.2 ± 0.8
Copper	4.5 ± 1.0
Iron	7.2 ± 1.4
Stainless Steel	6.6 ± 2.1
PMMA	2.6 ± 0.5
PET	2.9 ± 0.4
PVC	6.2 ± 0.7
Wood	8.0 ± 1.3
